# RObotic-Assisted Rehabilitation for balance and gait in Stroke patients (ROAR-S): study protocol for a preliminary randomized controlled trial

**DOI:** 10.1186/s13063-022-06812-w

**Published:** 2022-10-12

**Authors:** Silvia Giovannini, Chiara Iacovelli, Fabrizio Brau, Claudia Loreti, Augusto Fusco, Pietro Caliandro, Lorenzo Biscotti, Luca Padua, Roberto Bernabei, Letizia Castelli

**Affiliations:** 1 Department of Geriatrics and Orthopaedics, Università Cattolica del Sacro Cuore, Largo Francesco Vito, 8, 00168 Rome, Italy; 2 UOS Riabilitazione Post-Acuzie, Fondazione Policlinico Universitario A. Gemelli IRCCS, 00168 Rome, Italy; 3 Department of Aging, Neurological, Orthopaedic and Head-Neck Sciences, Fondazione Policlinico Universitario A. Gemelli IRCCS, 00168 Rome, Italy; 4 UOC Neuroriabilitazione Ad Alta Intensità, Fondazione Policlinico Universitario A. Gemelli IRCCS, 00168 Rome, Italy; 5 UOC Neurologia, Fondazione Policlinico Universitario A. Gemelli IRCCS, 00168 Rome, Italy; 6 Geriatric Care Promotion and Development Centre (C.E.P.S.A.G), Università Cattolica del Sacro Cuore, Rome, Italy

**Keywords:** Older adults, Elderly, Rehabilitation, Falls, Technology

## Abstract

**Background:**

Stroke, the incidence of which increases with age, has a negative impact on motor and cognitive performance, quality of life, and the independence of the person and his or her family, leading to a number of direct and indirect costs. Motor recovery is essential, especially in elderly patients, to enable the patient to be independent in activities of daily living and to prevent falls. Several studies have shown how robotic training associated with physical therapy influenced functional and motor outcomes of walking after stroke by improving endurance and walking strategies.

Considering data from previous studies and patients’ needs in gait and balance control, we hypothesized that robot-assisted balance treatment associated with physical therapy may be more effective than usual therapy performed by a physical therapist in terms of improving static, dynamic balance and gait, on fatigue and cognitive performance.

**Methods:**

This is an interventional, single-blinded, preliminary randomized control trial. Twenty-four patients of both sexes will be recruited, evaluated, and treated at the UOC Rehabilitation and Physical Medicine, Fondazione Policlinico Universitario A. Gemelli IRCCS in Rome from January to December 2022. Patients will be randomized into two groups: the experimental group will perform specific rehabilitation for balance disorder using the Hunova® robotic platform (Movendo Technology srl, Genoa, IT) for 3 times a week, for 4 weeks (12 total sessions), and for 45 min of treatment, in addition to conventional treatment, while the conventional group (GC) will perform only conventional treatment as per daily routine. All patients will undergo clinical and instrumental evaluation at the beginning and end of the 4 weeks of treatment.

**Conclusions:**

The study aims to evaluate the improvement in balance, fatigue, quality of life, and motor and cognitive performance after combined conventional and robotic balance treatment with Hunova® (Movendo Technology srl, Genoa, IT) compared with conventional therapy alone. Robotic assessment to identify the most appropriate and individualized rehabilitation treatment may allow reducing disability and improving quality of life in the frail population. This would reduce direct and indirect social costs of care and treatment for the National Health Service and caregivers.

**Trial registration:**

ClinicalTrials.gov NCT05280587. Registered on March 15, 2022.

**Supplementary Information:**

The online version contains supplementary material available at 10.1186/s13063-022-06812-w.

## Administrative information

Details of the sponsor, Funders, and their role in the study are listed in Table 1.

**Table 1:** Administrative information

Title	RObotic-Assisted Rehabilitation for balance and gait in Stroke patients (ROAR-S): Study protocol for a randomized controlled trial
Trial registration	ClinicalTrials.gov Identifier: NCT05280587
Protocol version	Protocol version number 2, date 7 December 2021
Funding	The trial did not receive any funding
Author details	1 Department of Geriatrics and Orthopaedics, Università Cattolica del Sacro Cuore, 00,168 Rome, Italy; 2 UOS Riabilitazione Post-acuzie, Fondazione Policlinico Universitario A. Gemelli IRCCS, 00,168 Rome, Italy; 3 Department of Aging, Neurological, Orthopaedic and Head-Neck Sciences, Fondazione Policlinico Universitario A. Gemelli IRCCS, 00,168 Rome, Italy; 4 UOC Neuroriabilitazione ad Alta Intensità, Fondazione Policlinico Universitario A. Gemelli IRCCS, 00,168 Rome, Italy; 5 UOC Neurologia, Fondazione Policlinico Universitario A. Gemelli IRCCS, 00,168 Rome, Italy; 6 Geriatric Care Promotion and Development Centre (C.E.P.S.A.G), Università Cattolica del Sacro Cuore, Rome, Italy
Name and contact Information for the trial sponsor	Fondazione Policlinico Universitario A. Gemelli IRCCS, Rome, Italy Main phone number: + 39 06 30 15 43 82
Role of sponsor	The contents of the published materials are the sole responsibility of the Sponsor, the Fondazione Policlinico Universitario A. Gemelli IRCCS, and the individual authors identified. The Sponsor will have no role in the study design, collection, data analysis and interpretation, or dissemination of the results, which will be the responsibility of the researchers

## Background and rationale

Stroke represents the second leading cause of disability worldwide; its incidence increases with age and around 25% of stroke affected people over 65 years old [1]. Stroke consequences, especially in older adults, affected one or more activities of daily living [2] and largely affect the quality of life and independence of the person and of his/her family. Moreover, stoke outcomes imply large direct and indirect social costs [3].

Motor recovery is essential in patients with stoke outcome [4, 5]; especially in older adults, a recovery of smoother, safer, and more correct walking is an essential requirement to allow the patient to be autonomous in the activities of daily living; trunk control and lower limb motor control are an essential outcome in stroke rehabilitation to prevent falls [6–8]. This is particularly important in this fragile population characterized by multimorbidity, polymedication, and nutritional deficit [9–11]. Several studies and a recent meta-analysis have shown how robotic training associated with physical therapy has influenced functional and motor gait and cognitive outcome after stroke, improving endurance and walking strategies [12–15]. In addition, frequently, stroke causes an impairment of the cognitive function that could affect the deterioration of balance and gait during dual-task activities; the study of these processes can be of interest for rehabilitation purposes [16–20]. Also important in rehabilitation are the motor and cognitive substrates that characterize each patient, which can positively influence the recovery process [21–23].

Considering data of the previous study and patients’ needs in the control of walking and balance, we have hypothesized a robotic-assisted balance treatment associated with physical therapy may be more effective than usual therapy performed by a physical therapist. Therefore, the present study aims to evaluate the effects of technological rehabilitation utilizing a robotic balance platform in terms of improvement in static, dynamic balance, and ambulation (assessed with clinical scales and instrumental measures) on fatigue and cognitive performance (attention, dual-task cost and cognitive-motor interference and on quality of life).

### Objective

The present study aims to evaluate the effects of a technological rehabilitation treatment and to assess fatigue and cognitive performance of treated patients.

### Trial design

The present study is an interventional, single-blinded, non-inferiority, randomized control pilot trial. The study protocol follows the Standard Protocol Items: Recommendations for Interventional Trial (SPIRIT) checklist (Additional file 1). The study registration data are shown in Table 1.

## Methods

### Study setting

**Fig. 1:**
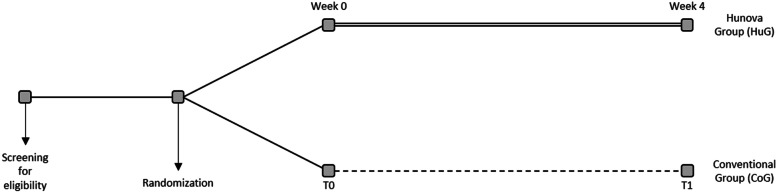
Study design with Group randomization

**Fig. 2:**
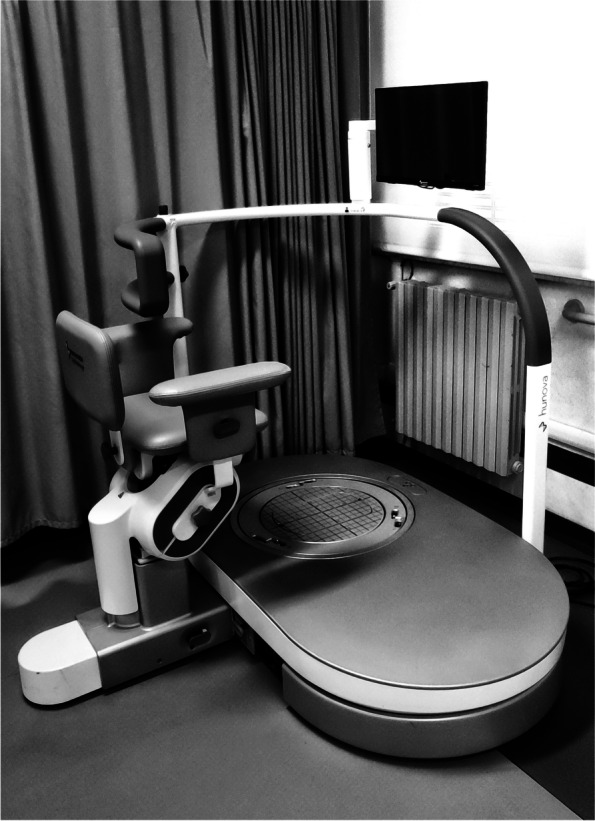
Robotic platform Hunova® (Movendo Technology srl, Genova, Italy) in our laboratory

**Fig. 3:**
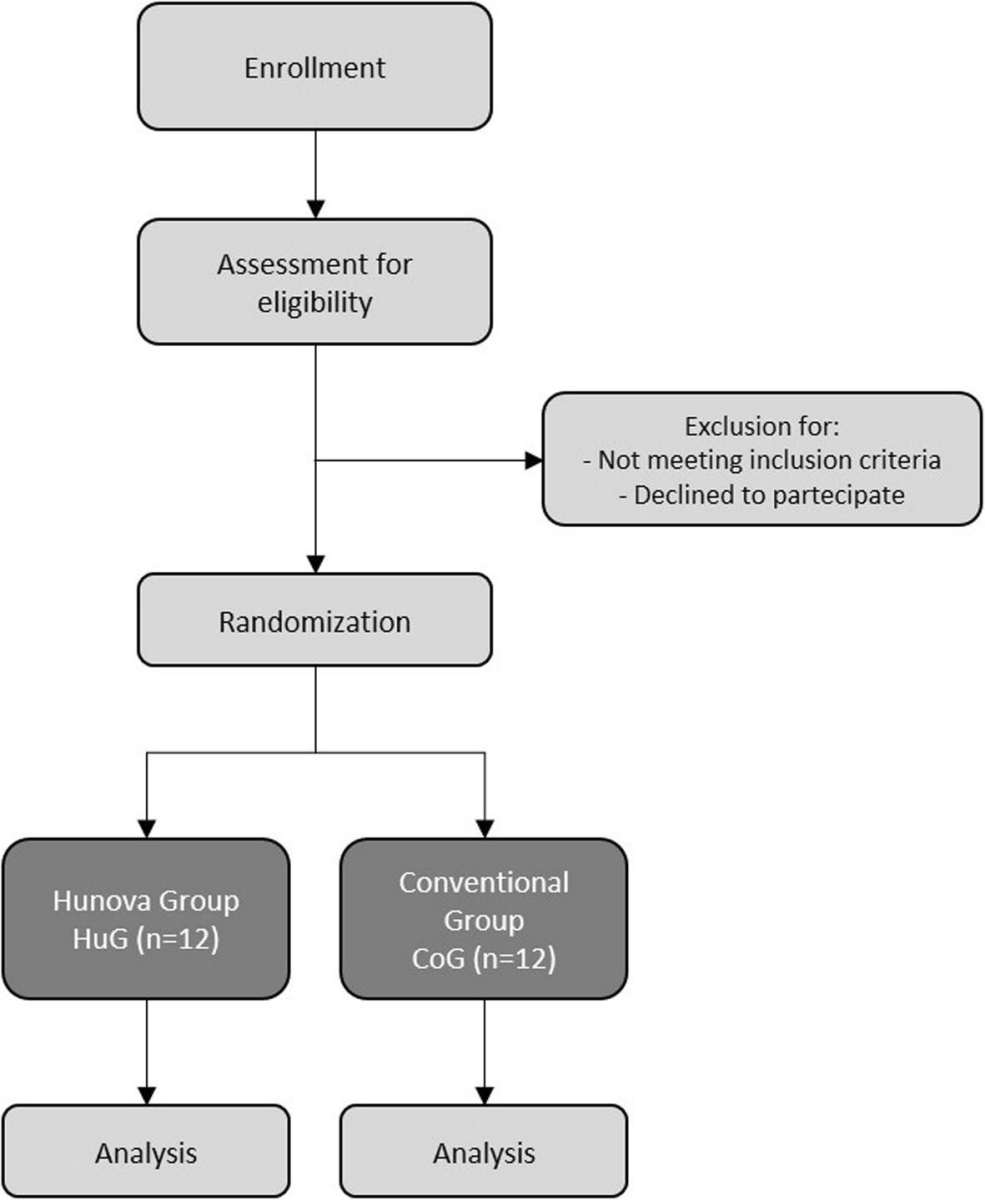
The study flowchart

The trial will be carried out at the Rehabilitation and Physical Medicine Unit of the Fondazione Policlinico Universitario A. Gemelli IRCCS in Rome from February 2022 to January 2023. It will include older adults with stroke outcomes of both sexes, who will be divided into two groups by randomization (Fig. 1): one experimental group (Hunova group, HuG) will perform specific rehabilitation for the balance disorder using the robotic platform Hunova® (Movendo Technology srl, Genova, IT; Fig. 2) 3 times a week, for 4 weeks (12 sessions), with each treatment lasting 45 min, in addition to the conventional treatment, and one group will perform only the conventional treatment (conventional group, CoG), as per daily routine, as described later. Figure 3 shows the study flowchart.

### Eligibility criteria

Elderly patients (age > 65 years) with ischemic or hemorrhagic stroke outcomes documented through neuroimaging techniques (magnetic resonance imaging or computed tomography) that occurred between 1 and 6 months previously will be included in the study, who have sufficient cognitive abilities to execute simple orders and to understand the physical therapist’s instructions as assessed through the Token Test (score ≥ 26. 5), who are able to walk independently or with minimal assistance, and who are able to understand and sign informed consent.

Patients with systemic, neurological, or cardiac pathologies that make ambulation risky or cause motor deficits, orthopedic or postural problems, plantar ulcers, and partial or total amputation of foot segments will be excluded.

### Who will take informed consent

Investigators participating in the study, through a comprehension test (see eligibility criteria), will screen patients to assess their inclusion in the study.

Participation in the study is voluntary: each patient will receive explicit information about the nature of the project and must sign a written consent before being included. Participants may withdraw consent to participate at any time without consequence.

In the event that the patient is not fully able to give informed consent due to disease characteristics, the family caregiver will be involved in the information and consent process along with the patient. Finally, researchers will collect signed informed consent, as required by the Ethics Committee.

### Assessment and intervention

#### Assessment

All participants declared eligible for the study will be evaluated at baseline (T0) and at the end of 4 weeks of treatment (T1).

At T0, researchers will collect demographical and clinical information of enrolled patients, such as age, sex, schooling, comorbidity, date of the event, etiology of the event, latency from the event, and pharmacological therapies in place. In addition, information on motor reserve, using the Lifetime Total Physical Activity questionnaire (LTPAq), and cognitive reserve, using the Cognitive Reserve Index questionnaire (CRIq), will be collected.

In addition to this, clinical and instrumental assessments will be conducted at both T0 and T1. Clinical assessment will be performed using the following rating scales: (i) Motricity Index Lower Limb (MI-LL) [24], Berg Balance Scale (BBS) [25], Time Up & Go (TUG) [26], and Short Physical Performance Battery (SPPB) [27] will be used to assess motor performance and balance; (ii) Ambulation Index (AI) [28], Walking Handicap Scale (WHS) [29], Functional Ambulation Classification (FAC) [30], the 10-Meter Walking Test (10MWT) [31], and 6-Minute Walking Test (6MWT) [32] will be used to assess ambulation performance; (iii) Modified Barthel Index (BIM) [33] and EuroQoL5D (EQ-5D) [34] will be used to assess autonomy and quality of life; (iv) Modified Fatigue Impact Scale (MFIS) [35] and Fatigue Scale for Motor and Cognitive Function (FSMC) [36] will be used to assess fatigue; (v) Frontal Assessment Battery (FAB) [37], Stroop Color Word Test (SCWT) [38], Symbol Digit Modalities Test (SDMT) [39], Digit Cancellation Test (DCT) [40], and Trail Making Test (TMT) [39] will be used to assess cognitive performance.

Instrumental assessment will include balance and gait analysis. Balance assessment will be carried out using the robotic platform (Hunova®, Movendo Technology srl, Genova, IT) and will be performed in static, dynamic, and dual-task conditions (i.e., during the execution of a concurrent cognitive task—the SCWT), both in sitting and standing positions. Posturography data will be obtained from the analysis of the trajectories of the center of pressure (CoP). Then, from the instantaneous CoP positions, we will obtain the following variables related to the balance performance: speed of the oscillations of the CoP along the anteroposterior (AP) and mid-lateral (ML) axes, length of the CoP trajectory, area of the 95% confidence ellipse, and Romberg Test [the ratio of the value of the length in the closed eyes (CE) condition and the same value in the open eyes condition (OE)].

Gait analysis will be performed using the Smart D500 stereo-photogrammetric system composed of 8 cameras, placing 22 markers on anatomical landmarks, according to as established by the Davis. The evaluation will involve the acquisition of 10 walking trials. Alongside a physical therapist, the patient will walk barefoot along a path of approximately 6 m at his/her natural speed. Before the actual assessment begins, the patient will be able to perform tests to familiarize himself or herself with the procedure and will be allowed to rest at the end of each trial. The gait analysis will be quantified in terms of spatial–temporal parameters of the gait cycle, joint kinematics, and range of motion (ROM) of the lower limb joints (hip, knee, and ankle), joint dynamics in terms of moments and powers, and ground reaction forces (GRFs).

Only at T1, each patient of HuG will complete the Technology Acceptance Model questionnaire (TAM) [41], to assess technology acceptance.

#### Intervention group

HuG patients will undergo robotic treatment for the improvement of balance through the robotic platform (Hunova® Movendo Technology srl, Genova, IT), 3 times per week, each lasting 45 min, in addition to the conventional treatment. In particular, the technological rehabilitation performed employing a footboard will be mostly aimed at improving the balance both in sitting and standing positions and will be proposed static and dynamic exercises, dual-task exercises, and exercises to improve trunk control.

#### Control group

CoG patients will undergo conventional rehabilitation treatment only, using the main rehabilitation methods (e.g., neurocognitive theory, Bobath concept, progressive neuromuscular facilitation). Specifically, activities for improving balance, coordination, walking, and overall motor and cognitive performance will be carried out.

#### Criteria for discontinuation or modification of assigned interventions

The physical therapists involved in the study will modulate rehabilitation activities according to the patient’s individual tolerance. This tailoring of treatment will be done to avoid problems arising from pain or fatigue.

#### Strategies to improve adherence to interventions

Adherence to rehabilitation interventions will be monitored by the treating physical therapists for both HuG and CoG.

#### Relevant concomitant care permitted or prohibited during the trial

There are no restrictions on concomitant care.

#### Provisions for post-trial care

No assistance is provided at the end of the trial.

### Outcome

#### Primary outcome

The primary outcome for motor performance is the BBS value, both in HuG and CoG, at the end of the 4 weeks of treatment.

### Secondary outcomes

Balance, ambulation, and motor performance will be assessed through MI-LL, TUG, SPPB, AI, WHS, FAC, 10MWT, and 6MWT. Compared with T0, the change after 4 weeks in disability, perceived quality of life, and fatigue was assessed by BIM, EQ-5D, MFIS, and FSMC. Cognitive performance will be assessed by FAB, SCWT, SDMT, DCT, and TMT.

### Participant timeline

Table 2 shows the timeline of data collection.

**Table 2:** Study plan and timing of procedures

Activity	Screening and enrolment	Week 0 (T0) Baseline	Week 4 (T1) Post-training
Token Test	X		
Signed informed consent	X		
Demographic and clinical data collection		X	
LTPAq		X	
CRIq		X	
MI-LL		X	X
BBS		X	X
TUG		X	X
SPPB		X	X
AI		X	X
WHS		X	X
FAC		X	X
10MWT		X	X
6MWT		X	X
Posturography (single-task and dual-task)		X	X
Gait analysis		X	X
BIM		X	X
EQ-5D		X	X
MFIS		X	X
FSMC		X	X
FAB		X	X
SCWT		X	X
SDMT		X	X
DCT		X	X
TMT		X	X
TAM			X

*LTPAq* Leisure Time Physical Activity questionnaire, *CRIq* Cognitive Reserve Index questionnaire, *MI-LL* Motricity Index Lower Limb, *BBS* Berg Balance Scale, *TUG* Timed Up & Go Test, *SPPB* Short Physical Performance Battery, *AI* Ambulation index, *WHS* Walking Handicap Scale, *FAC* Functional Ambulation Index, *10MWT* 10-Meter Walking Test, *6MWT* 6-Minute Walking Test, *BIM* Modified Barthel Index, *EQ-5D* Euro Quality of Life 5D, *MFIS* Modified Fatigue Impact Scale, *FSMC* Fatigue Scale for Motor and Cognitive Function, *FAB* Frontal Assessment Battery, *SCWT* Stroop Colour Word Test, *SDMT* Symbol Digit Modalities Test, *DCT* Digit Cancellation Test, *TMT* Trial making test, *TAM* Technology Acceptance Model questionnaire

### Sample size

Since this is a study of a specific subgroup of patients, on whom the actual utility of the Hunova® has not yet been studied in the literature, it is not necessary to formally estimate a minimum sample size. However, based on Julious’ rules [42], we estimate to enroll a sample of 24 subjects, randomized into two groups of equal size.

### Recruitment

Researchers, for eligibility, will evaluate all elderly people with stroke outcomes admitted to the post-acute rehabilitation unit.

### Assignment of interventions: allocation

Patients will be divided into two groups by randomization: one group will perform specific rehabilitation for balance disorder using the robotic platform (experimental group, HuG) in addition to conventional treatment, and the other group will perform only the conventional treatment (conventional group, CoG), as described later. The division into two groups will follow a randomization algorithm according to the procedure of random sorting. The allocation sequence will be generated through the PASS2019 software.

### Concealment method

The operator in charge of patient’s assessment will be blind to the allocation group, the operator applying the treatment will not be involved in the evaluation of patients, and a third operator will be responsible for generating the assignment sequence to the two groups.

### Implementation

After agreeing to participate, patients will be assigned an alphanumeric code to maintain data anonymity. The rehabilitation treatment physical therapist will be responsible for disclosing to the patient the group (HuG or CoG) to which they have been assigned.

### Assignment of interventions: blind

#### Who will be in the blinded

Blinding is limited to the researcher who will conduct the assessment and the researcher who will do the statistical analysis. Because of the visibility of the intervention, patients cannot be blinded.

#### Unblinding

Not applicable.

### Data collection and management

#### Plans for assessment and collection of outcomes

Researchers will collect data at the beginning and end of the 4-week treatment period. In addition, researchers will be trained in the administration of clinical scales.

#### Plans to promote participant retention and complete follow-up

In this study, there is no follow-up period after the end of the treatment period. In case participants discontinue the study, only data on the reason for discontinuation will be kept.

#### Data management

The study data will be collected and managed using the REDCap (Research Electronic Data Capture) electronic data capture tools [43, 44]. REDCap is a secure, Web-based software platform designed to support data capture for research studies by providing:

1. An intuitive interface for validated data capture
2. Audit trails for monitoring data manipulation and export procedures
3. Automated export procedures for downloading data into common statistical packages
4. Procedures for data integration and interoperability with external sources

Only persons officially registered as experimenters or data managers will receive a user login to access the REDCap web platform and enter/manage data.

#### Confidentiality

Participants will be assigned an alphanumeric identification code, and participant data will be stored on secure servers in accordance with national laws. It is planned to set up an operational database in which to enter information, drawn from the assessment protocol, deemed to be of priority importance. Data will be accessible only to investigators participating in the trial.

#### Plans for collection, laboratory evaluation, and storage of biological specimens for genetic or molecular analysis in this trial/future use

Not applicable.

### Statistical methods

#### Statistical methods for primary and secondary outcomes

The sample will be described in its clinical and demographic variables through techniques of descriptive statistics. The normality of the quantitative data will be checked with the Shapiro–Wilk test. Normally distributed quantitative variables will be summarized with the mean and standard deviation (SD) or the median and interquartile range (IQR), otherwise. Qualitative variables will be presented through absolute and percentage frequency tables. The primary objective, i.e., assessment of balance as measured by the increase in values of the BBS between start and end of treatment in the two groups, will be assessed employing an ANCOVA model, where the BBS value at baseline will be shown as a covariate, the BBS at the end of treatment as a dependent variable, and the treatment as a factor. The variations of the different parameters between the two groups at individual time instants will be evaluated, concerning the qualitative variables, by Fisher’s exact test or chi-square test, with Yates correction, where appropriate. Quantitative variables will instead be analyzed utilizing the Student’s *t*-test, in case of normally distributed data, or with the Mann–Whitney *U* test, in the opposite case. The assessment, among secondary endpoints, of fatigue, motor and cognitive performance, quality of life, ambulation quality of life, gait, and balance, and cognitive-motor interference will also be assessed through ANCOVA models, as previously indicated for the primary endpoint. Values of *P* < 0.05 will be considered statistically significant. All analyses will be conducted with R software version 4.1.1. (CRAN ®) and STATA version 16 (STATA Corp).

#### Interim analysis

No interim analysis is provided.

#### Methods for additional analysis

No additional analysis is provided.

#### Methods in analysis to handle protocol non-adherence and any statistical methods to handle missing data

Non-inferiority will be tested using two sets of analyses: the intention-to-treat set, which considers all patients as randomized regardless of whether they received the randomized treatment, and the “per-protocol” set of analyses. Missing values, all < 10%, were treated by *imputeR* package, within R Software version 4.2.0 (CRAN®, R Core 2022, Vienna, Australia) [45], using multiple imputation with Lasso Regression methods centered on the mean as for quantitative data, while classification trees for imputation by “rpartC” function, centered on the mode, i.e., most represented class object, were applied on qualitative data [46].

#### Plans to give access to the full protocol, participant-level data, and statistical code

Since this is experimental data, it will later be published and shared in an aggregated form that cannot be traced back to the individual participant with the national and international scientific community.

### Supervision and monitoring

#### Composition of the coordination center and steering committee for the trial

Researchers involved in the study will be responsible for enrollment, data collection, and treatment of patients. The research team is involved by a senior researcher and will plan monitoring meetings as needed.

#### Data monitoring committee composition, role, and reporting structure

A data monitoring committee was not considered relevant. The rehabilitation treatments that will be compared are feasible for patients and consist mainly of standardized exercise and robotic rehabilitation for balance improvement.

#### Harms

Given the nature of the rehabilitation interventions under study, no harm is expected.

#### Frequency and plans for verification of the conduct of the study

Verification will be conducted on a daily basis and at the end of the 4 weeks.

#### Plans for communication of major protocol amendments to relevant parties

Decisions on important changes in the trial, if they are necessary, will be made by the research team and authorized by the ethics committee.

### Dissemination plans

Results will be disseminated through peer-reviewed journals.

## Discussion

The study aims to evaluate the improvement in balance, fatigue, quality of life, and motor and cognitive performance after combined conventional and robotic balance treatment with Hunova® (Movendo Technology srl, Genoa, IT) compared with conventional therapy alone. Treatment efficacy will be defined based on motor outcomes (BBS, MI-LL, TUG, SPPB, AI, WHS, FAC, 10MWT, 6MWT) and cognitive outcomes (FAB, SCWT, SDMT, DCT, TMT) and for disability (BIM), quality of life (EQ-5D), and fatigue (MFIS, FSMC) by performance tests and specific self-administered questionnaires.

A possible limitation of the present study is the “non-early” treatment: in fact, the patient cannot undergo this balance-specific robotic treatment if he/she is unable to stand safely; therefore, the treatment cannot start in the acute phase. The considered period of the rehabilitative treatment, evaluated at 4 weeks (12 sessions), is sufficient to assess the feasibility of the protocol, but follow-up is needed to estimate the retention of maintenance of outcome achievement. Further studies will be conducted to determine these aspects and to evaluate the effectiveness of the treatment in other diseases.

A study on the rehabilitation of the frail population is of particular interest because of the continuing increase in older adults in Western countries. Robotic-assisted assessment to identify the most appropriate and individualized rehabilitation treatment may make it possible to reduce disability and improve the quality of life in this population. This study could allow the identification of tools that are effective for both motor and cognitive improvement to identify new strategies to counter the progression of disability and improve the daily management of elderly patients.

This would reduce direct and indirect social costs, of care and treatment, for the National Health Service and caregivers.

## Trial status

Protocol version 2, 7 December 2021. Initiation of recruitment of patients by February 2022. Enrollment is still ongoing. https://clinicaltrials.gov/ct2/show/NCT05280587?term=NCT05280587&draw=2&rank=1

## Supplementary Information

**Additional file 1.** SPIRIT Checklist.

**Additional file 2.** Informed consent.

## Data Availability

The research team will have full access to all data. The protocol and other research materials will be available upon reasoned request.

## Abbreviations

10MWT: 10-Meter Walking Test
6MWT: 6-Minute Walking Test
AI: Ambulation Index
AP: Anteroposterior
BBS: Berg Balance Scale
BIM: Modified Barthel Index
CE: Closed eyes
CoG: Conventional group
CoP: Center of Pressure
CRIq: Cognitive Reserve Index questionnaire
DCT: Digit Cancellation Test
EQ-5D: EuroQoL5D
FAB: Frontal Assessment Battery
FAC: Functional Ambulation Index
FSMC: Fatigue Scale for Motor and Cognitive Function
GRFs: Ground Reaction Forces
HuG: Hunova Group
IQR: Interquartile range
LTPAq: Lifetime Total Physical Activity questionnaire
MFIS: Modified Fatigue Impact Scale
MI-LL: Motricity Index- Lower Limb
ML: Mid-Lateral
OE: Open eyes
REDCap: Research Electronic Data Capture
ROM: Range of Motion
SCWT: Stroop Color Word Test
SD: Standard Deviation
SDMT: Symbol Digit Modalities Test
SPPB: Short Physical Performance Battery
TAM: Technology Acceptance Model questionnaire
TMT: Trial Making Test
TUG: Timed Up & Go
WHS: Walking Handicap Scale
