# Supplementary material for: RObotic-Assisted Rehabilitation for balance and gait in Stroke patients (ROAR-S): study protocol for a preliminary randomized controlled trial
Source: Trials. 2022 Oct 12;23:872. doi: 10.1186/s13063-022-06812-w (PMC9558956; doi:10.1186/s13063-022-06812-w)
Supplement: Supplementary file 2 — Additional file 2. Informed consent. [file 13063_2022_6812_MOESM2_ESM.pdf]

INFORMED CONSENT FOR PARTECIPATION IN A CLINICAL TRIAL AND STATMENT OF CONSENT

For an adult patient capable of giving personal content

Version 1.1; 23 november 2021

Title: **RObotic Assisted Rehabilitation for balance and gait in Stroke patients (ROAR-S):**

Dear Madam/Dear Sir,

the following information is very detailed. We ask that you agree to participate in the trial ONLY after reading this sheet carefully and having an extremely detailed interview with a member of the trial team.

It is your right to be informed about the purpose and characteristics of the trial so that you can make a free and informed decision whether or not to participate.

The purpose of this document is to inform you about the nature of the trial, its purpose, and what participation will entail for you. Please read the following carefully. The researchers involved in this project are available to answer your questions.

You may take an unsigned copy of this document home to think about it or to discuss it with others before making a decision.

If you decide not to participate in the study, you will still receive the best possible care for patients with your condition/disease.

Your refusal will in no way be interpreted as a lack of trust.

Principal Investigator

INFORMATION SHEET

Dear Madam/Dear Sir,

A study entitled " RObotic Assisted Rehabilitation for balance and gait in Stroke patients (ROAR-S)" is being planned at the Policlinico Universitario A. Gemelli-IRCCS.

This research is nationwide, single-center and aims to test a new rehabilitation procedure for the care of patients suffering from the same disease from which you are affected.

In order to carry out this research, we would like to take advantage of the cooperation and availability of people who, like you, meet the appropriate scientific requirements for the evaluation that will be carried out. Your decision whether or not to participate in this study will have no impact on the care you receive, and the doctors will continue to follow you with due care.

Before making your decision to accept or decline participation, however, we encourage you to read these pages carefully, taking your time, and to ask for clarification if you do not fully understand or need further elucidation. In addition, if you wish, you may seek advice from your family members or a trusted physician before making your decision.

WHAT THE STUDY PROPOSES

The overall objective of the study is to evaluate the benefits to the patient after performing a rehabilitation treatment using a robotic platform. Specifically, this study aims to evaluate the effects of this treatment on balance, gait, fatigue, cognitive component, and quality of life.

The experimental design calls for patients to be randomly assigned to either the experimental group or the control group. Patients in the experimental group, in addition to the rehabilitation provided by the rehabilitation design, will undergo specific balance and gait rehabilitation via the Hunova® robotic platform 3 times a week for 4 weeks (12 total sessions) for 45 minutes. Patients in the control group, on the other hand, will undergo the usual rehabilitation project provided by clinical practice.

If you agree to participate in this study, you will undergo an initial visit to verify that your condition meets the criteria required for participation. At this visit, the "token test" will be performed to assess comprehension.

If you are found to be eligible to participate in the study, you may undergo 2 additional visits, which will be conducted before you begin rehabilitation treatment with the robotic platform and at the end of the 12 treatment sessions. During these meetings, he/she will be assessed through clinical scales for static and dynamic balance, ambulation, autonomy, quality of life, fatigue, and cognitive performance. In addition, he or she will also undergo instrumental assessment of balance, using the Hunova® robotic platform, and gait, by performing a gait analysis, also on these occasions.

The study will last 4 weeks, and 24 patients with the same disease as him will participate in the research at this hospital.

Participation in the trial involves no user fees and no compensation.

WHAT ARE THE RISKS OF PARTICIPATING IN THE STUDY

Participation in this study poses no major risks to the patient, as no invasive treatments or therapies are planned. In addition:

- (i) the procedures used for clinical and instrumental assessment are tools widely used in clinical practice and pose no special risks;
- (ii) during both Hunova® treatment and clinical and instrumental evaluations, the patient will be constantly supported and supervised by a physical therapist who can intervene promptly if necessary.

All procedures involved in the research will be carried out paying special attention to the patient involved, taking all necessary measures so that stress or fatigue-related criticalities do not occur.

WHAT ARE THE BENEFITS YOU MAY RECEIVE BY PARTICIPATING IN THE STUDY

The following benefits are expected from participation in this study: improved balance, walking, perception of fatigue, quality of life and cognitive performance.

STUDY RESULTS AND CONFIDENTIALITY OF INFORMATION COLLECTED

All of your data will be pseudo-anonymized, meaning that you will be assigned a code that is not directly traceable to you and will be recorded in a password-protected electronic format. This code will not allow you to be identified outside the treatment center.

With regard to the processing of personal data, you should refer to the specific notice for the manifestation of consent to the processing of personal data that will be delivered to you at the same time, on a separate sheet.
You are free to decide whether or not to be informed about these kinds of results.

WHAT HAPPENS IF YOU DECIDE NOT TO PARTICIPATE IN THE STUDY - ANY ALTERNATIVE TREATMENTS.

You are free not to participate in the study. In this case, you will still continue to receive all the standard therapies and treatments provided for your condition, without any penalty, and the doctors will continue to follow you with due care, even if no other therapies (experimental or otherwise) are available.

STUDY INTERRUPTION

Your participation in this research program is completely voluntary and you may withdraw from the study at any time by informing the investigator. In this case, data collected up to the time of withdrawal will not be considered in the results in aggregated and anonymized form for final analysis.

Similarly, the study may be terminated if:

(i) the physician does not observe a benefit or if side effects or otherwise have occurred;
(ii) new information becomes available and the trial is no longer in your best interest;
(iii) the patient has not complied with the agreed-upon rules for participation in the trial;
(iv) the study is stopped by the competent authorities or the sponsor.

In such cases, you will be promptly informed about further valid treatments for the disease you have, you can discuss them with your doctor, and in any case the center will continue to follow you with due attention, even if there are no other treatments available.

INFORMATION ON THE RESULTS OF THE STUDY

If you request, upon completion of the study, you may be notified of the results of the study in general and in particular those that concern you.

The proposed study protocol has been prepared in accordance with current revisions of the European Union's Standards of Good Clinical Practice and the World Medical Association's Declaration of Helsinki on Clinical Trials Involving Human Subjects and has been approved by the Ethics Committee of this facility. You may report any facts that you deem appropriate to point out, with regard to the trial that concerns you, to the Ethics Committee and the Health Management of this facility.

DECLARATION OF CONSENT

I DECLARE

▢ that I have received from Dr. ________________________ comprehensive explanations regarding the request to participate in the research in question, as stated in the information notice of which I was given a copy in advance, forming part of this consent, a copy of which was delivered to me on ____________;

▢ which were clearly explained to me and I understood the nature, purpose, procedures, expected benefits, possible risks and disadvantages, and alternative treatment modalities to the proposed clinical trial;

▢ to have had the opportunity to ask questions of the study researcher and having received satisfactory answers;

▢ to have had sufficient time to reflect on the information received;

▢ to have had sufficient time to discuss it with third parties;

▢ that I have been informed that the protocol of the trial and all the forms used have had the favourable opinion of the relevant Ethics Committee;

▢ to be aware that the search may be terminated at any time;

▢ that I have been informed that I will be made aware of any new data that may compromise the safety of the research and that, for any problems or further questions, I can contact the principal investigator or his/her collaborators;

▢ that for the best protection of my health I am aware of the importance of informing the general practitioner of the trial in which I agree to participate.

▢ that am aware of the importance of providing all information (medications, side effects, etc.) about me, to the investigator;

▢ that I have been informed that the results of the study will be released to the scientific community, protecting my identity according to current privacy regulations;

▢ to be aware that any choice expressed in this consent form may be revoked at any time without justification;

▢ that I have received a copy of this consent form.

Place and date ___________________________________

First and Last name of the patient ___________________________________

Signature of the patient ___________________________________

First and Last name of the
Researcher who collected the consent ___________________________________

Signature of the researcher who
collected the consent ___________________________________
